# Electroacupuncture decreases cognitive impairment and promotes neurogenesis in the APP/PS1 transgenic mice

**DOI:** 10.1186/1472-6882-14-37

**Published:** 2014-01-22

**Authors:** Xuying Li, Fan Guo, Qiaomei Zhang, Tingting Huo, Lixin Liu, Haidong Wei, Lize Xiong, Qiang Wang

**Affiliations:** 1 Department of Anesthesiology, Xijing Hospital, The Fourth Military Medical University, Xi’an 710032, Shaanxi Province, China; 2 Department of Anesthesiology, Stony Brook University School of Medicine, HSC L4 060, Stony Brook, NY 11794, USA

**Keywords:** Alzheimer’s disease, Electroacupuncture, Neurogenesis, BDNF, Aβ deposits

## Abstract

**Background:**

Alzheimer’s disease (AD) is a severe neurodegenerative disease for which there is currently no effective treatment. The purpose of this study was to investigate whether repeated electroacupuncture (EA) stimulation would improve cognitive function and the pathological features of AD in amyloid precursor protein (APP)/presenilin 1 (PS1) double transgenic mice.

**Methods:**

Cognitive function of APP/PS1 double transgenic mice was assessed using the Morris water maze test before and after EA treatment. Levels of amyloid β-peptide (Aβ) deposits in the hippocampus and cortex were evaluated by immunofluorescence, western blot and enzyme-linked immunosorbent assay. Expression of brain-derived neurotrophic factor (BDNF) was also examined by immunofluorescence and western blot. The neurogenesis was labeled by the DNA marker bromodeoxyuridine.

**Results:**

EA stimulation significantly ameliorated the learning and memory deficits of AD mice by shortening escape latency and increasing the time spent in the target zone during the probe test. Additionally, decreased Aβ deposits and increased BDNF expression and neurogenesis in the hippocampus and cortex of EA-treated AD mice were detected. The same change was detected in wild-type mice after EA treatment compared with wild-type mice without EA treatment.

**Conclusions:**

Repeated EA stimulation may improve cognitive function, attenuate Aβ deposits, up-regulate the expression of BDNF and promote neurogenesis in the APP/PS1 double transgenic mice. This suggests that EA may be a promising treatment for AD.

## Background

Alzheimer’s disease (AD) is one of the most common neurodegenerative diseases. Its main clinical manifestations include dementia, memory loss, personality disorders and language problems. The global prevalence of dementia was estimated to be as high as 24.2 million by 2005, and about 70% of these cases were attributed to AD [1]. Prevalence is predicted to reach 80 million worldwide by 2040 [2]. Confronted with such a large number of people suffering from AD, the currently available treatments for the disease are limited and without curative effects. Therefore, identifying effective and safe treatment with clear fundamental mechanisms is urgently needed.

The pathology of AD is generally accepted as being characterized by the abnormally abundant deposition of amyloid plaques, neurofibrillary tangles, and selective neuronal loss in the frontal and temporal cortices, as well as the hippocampus of brain. The accumulation of amyloid β-peptide (Aβ) plays the most important role in the pathogenesis of AD. A wealth of evidence has indicated that Aβ1-42 deposits participate in the process of neuron loss and lead to the occurrence of dementia in AD patients.

AD is a multifactorial disease, and a decrease in neuro-regenerative capacity is an important factor in the decline of neural plasticity, development of Aβ plaques and neurofibrillary tangles [3]. Numerous studies have provided powerful evidence that the decline of hippocampal neurogenesis participates in the development of AD and induces impairment of learning and memory [4]. Several studies have indicated that brain-derived neurotrophic factor (BDNF) is an important factor for promoting neurogenesis in the adult central nervous system under physiological or pathological conditions [5,6]. Moreover, some clinical studies have revealed that the level of BDNF expression is significantly decreased in the hippocampus and some cortical areas of AD patients [7,8]. These data suggest that up-regulation of BDNF and promotion of neurogenesis would be a promising target for AD treatment.

Electroacupuncture (EA), a traditional Chinese medicine treatment that stimulates certain acupoints, has been shown to induce significant neuroprotective effects in various kinds of central nervous system diseases, as well as improve neuroethology [9-11]. The mechanism for EA improving neurological deficits in ischemic injury is proven to be through the promotion of neurogenesis [12]. EA has also been proven to enhance BDNF activation in the dentate gyrus in rats [13]. However, whether EA stimulation is effective for AD still remains unclear.

In the present study, we investigated whether EA treatment could ameliorate cognitive impairment and attenuate Aβ deposits, and the effect of EA treatment on BDNF expression and neurogenesis in the amyloid precursor protein (APP)/presenilin 1 (PS1) double transgenic (Tg) mice.

## Methods

### Animals

We used an APP/PS1 double Tg C57BL/6 J mouse model, which could effectively simulate the pathological features of AD patients. We used this model to investigate the effect of EA treatment on AD mice. Male APP/PS1 double Tg mice (2-months old) were purchased from Beijing HFK Bioscience Co. LTD (Beijing, China) and randomly divided into two groups: an APP/PS1 group (APP) and an EA treatment APP/PS1 group (APP + EA). Normal male C57BL/6 J mice (2 months old) were obtained from the Experimental Animals Center of the Fourth Military Medical University (Xi’an, China) and randomly divided into two groups: a control group (Con) and an EA treatment control group (Con + EA). Animals were housed under controlled temperature (25°C), 12-hour light/dark cycles and allowed free access to water and food. All experiments were carried out according to the Guidelines for Animal Experimentation of the Fourth Military Medical University. The experimental protocol was approved by the Ethics Committee for Animal Experimentation, and was performed according to the Guidelines for Animal Experimentation of the Fourth Military Medical University and to the National Institute of Health Guide for the Care and Use of Laboratory Animals.

### EA treatment

In line with our previous studies, we performed EA treatment at the “Baihui (GV20)” acupoint with an intensity of 1 mA and frequency of 2/15 Hz for four weeks for total 20 days (30 min/day, 5 days/week) [10,14]. We used an EA instrument (Hwato, model No. SDZ-V, Suzhou Medical Instruments Co, Ltd, Suzhou, China) and selected the dense-sparse wave type. During EA treatment, we maintained the rectal temperature of all animals at 37.0 ± 0.5°C and we provided inhaled oxygen by facemask at a flow rate of 1 L/min. The animals in control group received no treatment.

### Morris water maze test

Following the methods previously described, we used the Morris water maze (MWM) test to evaluate learning and memory impairment in each group in this experiment. We evaluated the Morris water maze test at 7 months and 10 months (before EA treatment), and at 11 months (immediately after EA treatment). The apparatus consisted of a circular pool (120 cm diameter × 50 cm height) with a black inner wall, which was subdivided into four equal quadrants and filled with water (25°C) to the depth of 30 cm. An escape platform (10 cm diameter) was placed in one of the quadrants (the target quadrant) and submerged approximately 2 cm below the surface of the water. Mice were released into the water facing the wall of the pool. The test contained a platform trial that measured the animal’s spatial acquisition ability and a spatial probe test that assessed memory. On the first day, the mice in each group performed four platform trials with the platform submerged in water in the same place each time. They then performed four training trials per day for five days. Finally, 24 hours after the 5^th^ day, a probe test, where the platform was removed, was performed. All the data, including the swim path and the swim time, were measured by an automated analyzing system (Dig-Behav, Jiliang Co., Ltd., Shanghai, China).

### Sample preparation and double-label immunofluorescence assays

Immunofluorescence was used to evaluate levels of Aβ deposits and BDNF expression in the hippocampus and cortex of each group. At first, to remove the blood and fix the brain tissues, the Tg mice were deeply anesthetized with 0.8% pentobarbital sodium and perfused with physiological saline, followed by 4% (v/v) ice-cold paraformaldehyde in PBS (pH = 7.4). Brain tissues were then harvested and postfixed for 2 hours in the same fixative at 4°C and cryoprotected in 20% and 30% sucrose solutions. Coronal Sections 12 μm in thickness were cut using a cryostat and block with PBS containing 0.3% (v/v) Triton X-100 and 3% (v/v) normal goat serum. To verify the success of the AD model building, the tissue sections were incubated overnight with rabbit anti-Aβ primary antibodies (1:400, Cell Signaling Technology, Inc. Danvers, MA, USA) followed by the anti-rabbit Cy3 tagged secondary antibodies (1:200; CWBIO, Beijing, China). To investigate the effect of EA on Aβ deposits and BDNF expression in the brain, immunofluorescence was conducted. The sections were incubated with the first antibody of rabbit anti-BDNF (1:200, Beijing Biosynthesis Biotechnology Co., Ltd, Beijing, China) or rabbit anti-Aβ antibody (1:400, Cell Signaling Technology, Inc. Danvers, MA, USA) followed by the anti-rabbit Cy3 tagged secondary antibodies (1:200; CWBIO, Beijing, China). The sections were mounted with 50% glycerol for examination under a fluorescence microscope. Images were observed and captured with a co-focal laser microscope (FV1000; Olympus BX51; Tokyo, Japan).

### Western blot analysis

Animals of each group were anesthetized with sodium pentobarbital (100 mg/kg, i.p.) and sacrificed immediately by decapitation. Brain tissues were then removed, weighed and homogenized in RIPA buffer (Beyotime P0013C, Haimen, Jiangsu, China) plus protease inhibitors. A BCA Protein assay kit (Beyotime P0012S, Haimen, Jiangsu, China) was used to determine the protein concentrations of each sample; 40ug protein samples were separated by 15% SDS-PAGE. The proteins were then transferred onto polyvinylidene difluoride membrane. After blocking with 3% nonfat milk, membranes were incubated with the following primary antibodies: rabbit anti-BDNF monoclonal antibody (1:100, Beijing Biosynthesis Biotechnology Co., Ltd, Beijing, China), rabbit anti-β-actin (1:10000, Epitomics, Inc., P60709), and rabbit anti-Aβ (1:400, Cell Signaling Technology, Inc. Danvers, MA, USA). Subsequently, membranes were incubated for 1 hour at room temperature with secondary antibody of anti-rabbit HRP-conjugated IgG (1:20000, CWBIO, Beijing, China). Labeled protein was detected using chemiluminescence reagents (ECL; Amersham Bio-sciences, Little Chalfont, Buckinghamshire, UK) and the band intensity was analyzed (Alpha Innotech).

### Quantification of Aβ1-42 level by ELISA

Levels of soluble Aβ1-42 in the hippocampus and cortex of Tg mice were determined with sandwich enzyme-linked immunosorbent assay (ELISA), using mouse β-Amyloid (1–42) ELISA kit (Westang Ltd, Shanghai, China). According to the manufacturer’s instruction, brain tissues were homogenized in RIPA buffer (Beyotime P0013C, Haimen, Jiangsu, China) and centrifuged at 27,000 × g at 4°C for 30 min to obtain the supernatants. Then, the standards and samples which were mixed with specific first antibody in duplicate were added to the microtiter wells. HRP-conjugated secondary antibody was added to the plates for 1 h at room temperature after extensive washing, followed by chromogen for 15–30 min. The enzymatic reaction was then terminated by addition of a stop solution (2 N H_2_SO_4_). Optical density (OD) was read at 450 nm within 30 min on a microplate spectrophotometer (Denley Dragon Wellscan MK 3). Concentrations were calculated according to the standard curve.

### BrdU staining

The newly born neurons were labeled by DNA marker Bromodeoxyuridine (BrdU) (Sigma, St. Louis, MO, dissolved in 0.9% NaCl). BrdU was administered intraperitoneally at a concentration of 100 mg/kg/day by injection for seven consecutive days. And the first administration was at the last 2 rest day of 4^th^ cycle of the electroacupuncture. To label differentiated neurons in the hippocampus and cortex, 11-month-old Tg mice APP, APP + EA and nontransgenic mice Con, Con + EA (n = 5 each) were sacrificed 28 days after the last injection of BrdU. The mice were then anesthetized and perfused with phosphate-buffered saline following by 4°C 4% paraformaldehyde. The brain was removed and postfixed in buffered 4% paraformaldehyde for 2 hours. After being immersed in 20% and 30% sucrose solution, the brains were cut into 12 μm-thick coronal sections.

To label the relative distribution of the new proliferating cells, BrdU + Nissl double staining was used. First, sections of every group were incubated with 10% normal goat serum in 0.1 M PBS 60 min at 37°C. The primary antibodies Nissl (Abcam) and rat anti-BrdU (ab6326, Abcam LTD., Hongkong, China) were used. After washing in PBS, the sections were incubated in secondary antibody Biotinylated Goat Anti-body IgG antibody (1:300, Vector, BA 9400), and the third antibody Cy3-conjugeted streptavidin (1:800). For each group, brain sections starting from the bregma -2.1 and ending at the bregma -4.5 were selected for counting of the BrdU(+) and Nissl(+) cells. The BrdU(+) and Nissl(+) cells were calculated using a × 400 magnification in the DG, CA1 regions.

### Statistical analysis

SPSS 14.0 for Windows was used to conduct the statistical analyses. Two-way analysis of variance (ANOVA) with repeated measures was used for analyzing data from the Morris water maze test. Other statistical tests were performed using one-way ANOVA and Student’s t-test for comparisons. The *P* values of ≤ .05 were considered indicative of statistical significance. All date are expressed as mean ± standard deviation (SD).

## Results

### Cognitive impairment and Aβ_1-42_ deposition presented in the APP/PS1 mice

**Figure 1:**
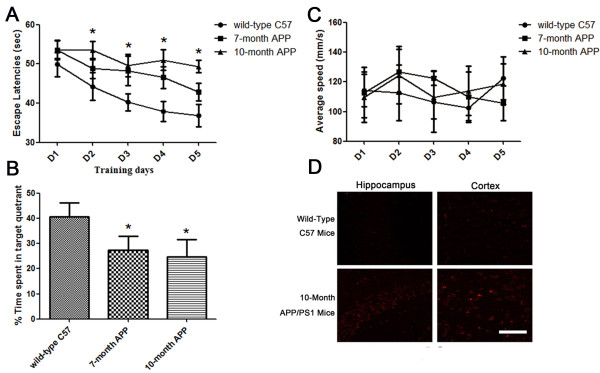
**APP/PS1 mice demonstrated features of cognitive impairment seven months after birth. (A)** 7-month-old and 10-month-old APP/PS1 mice showed longer latencies for reaching the platform in the Morris Water Maze test than the wild-type mice. **(B)** The average swimming speeds among different groups were not significant different. **(C)** During the probe trail, wild-type mice spent more time in the target quadrant than 7-month-old and 10-month-old APP mice. (n = 6 in each group) (*P < .05 from wild-type mice); **(D)** Compared with the wild-type mice, total Aβ deposits in the hippocampus and cortex of 7-month-old and 10-month-old APP mice were increased.

Transgenic mice, which imitated the most salient characteristics of AD, were selected to simulate human Alzheimer’s disease. The effectiveness of the Tg mouse model to mimic AD was evaluated using a neurological behavior test (Morris water maze test) and a pathology test (immunofluorescence that specifically identified a biomarker of AD) of 7-month-old and 10-month-old Tg mice. Spatial learning and memory function performance were more severely damaged in the 7-month-old group of double Tg mice than in the wild-type C57 mice; this was evaluated by the platform trial and spatial probe test of the Morris water maze test (Figure 1A, B) without a significant difference in swimming speed (Figure 1C). However, there was no significant difference between the 7-month-old and 10-month-old Tg mice groups. This indicated that the pathological features of APP/PS1 double Tg mice developed from at least 7 months and remained stable.

Total Aβ deposits in the hippocampus and cortex were detected by quantitative immunofluorescence in different groups (Figure 1D). The Aβ level of the hippocampus and cortex in the 10-month-old group was increased compared with wild-type group.

### EA ameliorated cognitive impairment in APP/PS1 double Tg mice

**Figure 2:**
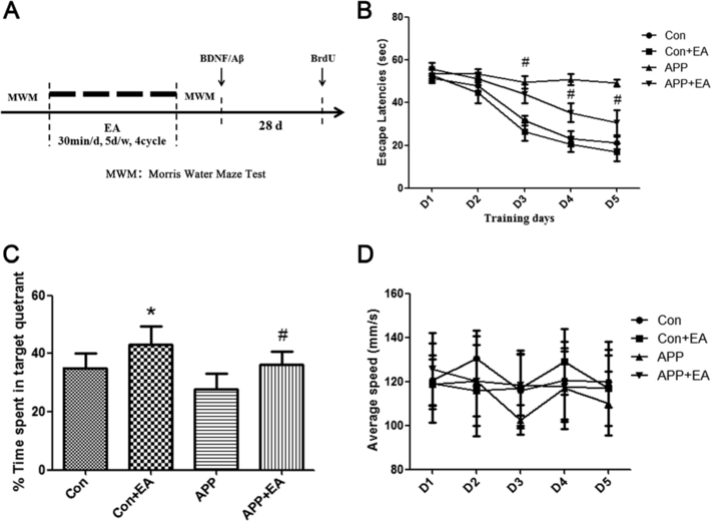
**Electroacupuncture ameliorated cognitive impairments in APP/PS1 mice. (A)** Schematic representation of the experiment protocol. **(B)** Mice from the APP group showed longer latencies for reaching the platform than those from the APP + EA group. **(C)** The probe test indicated that the time spent in the target quadrant was increased in the APP + EA group and Con + EA group compared with their non EA-treated counterparts. **(D)** No significant difference was detected in average swimming speed among all the groups. (n = 6 in each group) (*P < .05 vs. Con group; #*P* < .05 vs. APP group).

We investigated the effects of EA stimulation on cognitive function through use of the Morris water maze (MWM) test. This test was conducted following the procedure diagram shown in Figure 2A. As shown in Figure 2B-C, escape latency indicated that the APP + EA group had better cognitive performance than the APP group. Cognitive impairment in the different groups was confirmed in the probe trail, which showed that the APP + EA group and the Con + EA group mice spent more time in the target quadrant than their non EA-treated counterparts (*P* < .05). This difference was not attributable to the presence of motor deficits as the four groups of mice exhibited similar swimming speeds (Figure 2D). Taken together, the above results demonstrate that EA stimulation significantly improved learning and memory functioning in both APP/PS1 Tg mice and wild-type mice.

### EA reduced brain Aβ_1-42_ deposition in APP/PS1 double Tg mice

**Figure 3:**
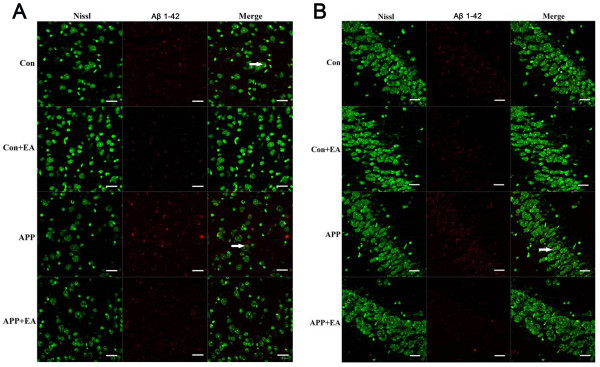
**Electroacupuncture suppressed the deposition of Aβ**_**1-42 **_**in the hippocampus and cortex.** With immunofluorescence staining following the EA administration, compared with the APP group, the deposition of Aβ1-42 was significantly decreased in the APP + EA group in the **(A)** cortex and **(B)** hippocampus. Bar = 20 μm.

To investigate the effect of EA on Aβ_1-42_ deposition in the hippocampus and cortex, three methods, including immunofluorescence staining, ELISA and western blots, were used. As shown in Figure 3A-B, Aβ_1-42_ staining (red) was co-localized with neurons (Nissl staining, green) in all studied regions. The APP + EA group exhibited notable reductions in Aβ_1-42_ deposits compared with the APP group, in both the hippocampus and cortex. Meanwhile, EA stimulation also reduced the deposit of Aβ_1-42_ in normal-aged mice compared with the Con group.

**Figure 4:**
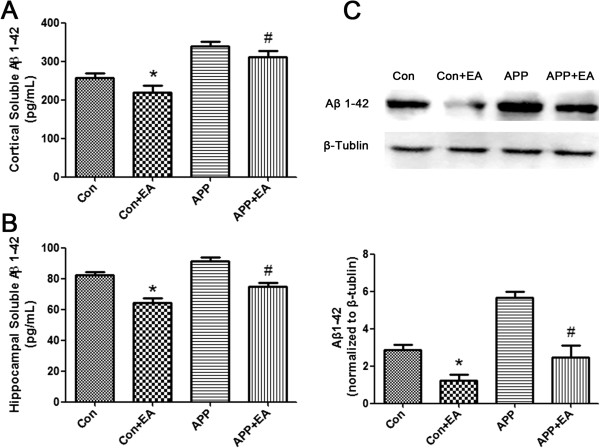
**Electroacupuncture suppressed the deposition of Aβ**_**1-42 **_**in the hippocampus and cortex.** ELISA test results revealed that Aβ1-42 depositions from the **(A)** cortex and **(B)** hippocampus in the APP + EA group and the Con + EA group were significantly lower than those in their non EA-treated counterparts (n = 5 in each group) (*P < .05 vs. Con group; #*P* < .05 vs. APP group). **(C)** Western blot result indicated that the expression of Aβ_1-42_ in the Con + EA group was lower than that in Con group. A similar result was detected in the APP + EA group compared with the APP group. (n = 3 in each group) (*P < .05 vs. Con group; #*P* < .05 vs. APP group).

ELISA test results showed that the levels of Aβ_1-42_ deposits from the hippocampus and cortex in the APP + EA group and the Con + EA group were significantly lower than those in their non EA-treated counterparts (*P* < .05; Figure 4A-B). At the same time, gray-scale analysis of western blot bands indicated that the expression of Aβ_1-42_ from the APP + EA group was lower than that in the APP group (Figure 4C-D). A similar result was detected in the Con group when compared with the Con + EA group. These results demonstrate that long-term EA treatment is an effective method for inhibiting the deposition of Aβ_1-42_.

### EA increased BDNF expression in APP/PS1 double Tg mice

**Figure 5:**
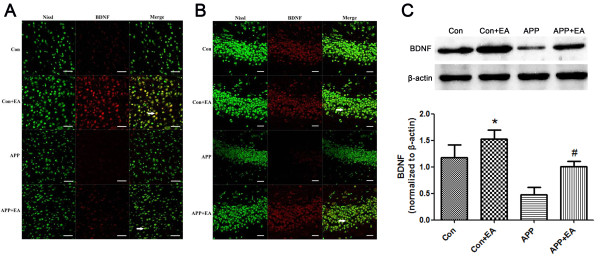
**Electroacupuncture upregulated the expression of BDNF in hippocampus and cortex.** Representative double immunofluorescence staining of BDNF (red) and NeuN (green) in the **(A)** cortex and **(B)** hippocampus. With EA treatment, BDNF was upregulated in the APP + EA group and Con + EA group compared with their non EA-treated counterparts (*P < .05 vs. Con group; #*P* < .05 vs. APP group). **(C)** The protein expression of BDNF was evaluated using western blot. EA significantly up-regulated the expression of BDNF in the APP/PS1 mice and wild-type mice compared with their matched counterparts. Data are means ± SD (n = 5 in each group) (*P < .05 vs. Con group; #*P* < 0.05 vs. APP group). Bar = 20 μm.

The results of immunofluorescence with the antibody specifically recognizing BDNF in the brain showed significantly stronger expression in both the hippocampus and cortex from the EA-treated groups than that in the non EA-treated groups (Figure 5A-B). Western blot results also confirmed that the expression of BDNF was up-regulated in the APP + EA and Con + EA groups compared with their non EA-treated counterparts (Figure 5C, *P* < .05).

### EA promoted neurogenesis in APP/PS1 double Tg mice

**Figure 6:**
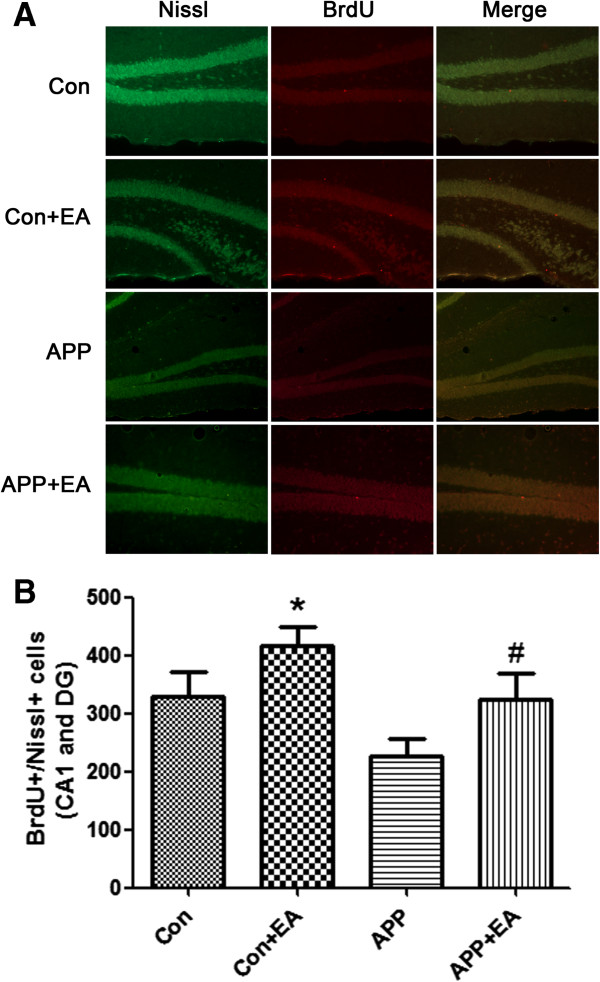
**Electroacupuncture enhanced neurogenesis in hippocampal region of APP mice. (A)** Representative immunofluorescence of BrdU (red) and Nissl (green) staining in the absence or presence of EA treatment. **(B)** Quantification of BrdU+/Nissl + cells in the CA1 and DG region. Data are presented as means ± SD (n = 5 in each group) (*P < .05 vs. Con group; #*P* < .05 vs. APP group).

Survival and differentiation of the newborn cells to neurons (BrdU + Nissl+) were detected 28 days after the last injection of BrdU. As shown in Figure 6, the result of BrdU(+) and Nissl(+) cells was consistent with the change of BDNF expression in the brain. There was a significant decrease in BrdU(+) and Nissl(+) cells in the APP/PS1 Tg mice groups (including the APP and APP + EA groups) when compared with the wild-type group (including Con and Con + EA groups) (*P* < .05). EA treatment also significantly promoted neurogenesis in the APP + EA group, as well as in the Con + EA group, compared with their non EA-treated counterparts.

## Discussion

AD is a neurodegenerative disease that seriously affects the quality of life for thousands of patients. There is also an enormous economic impact of AD for governments. In Europe, the total costs for dementia in 2008 were estimated to be more than 177 billion Euros [15], which exceeded the costs for patients with either cancer or cardiovascular diseases [2]. The existing treatments for AD are unsatisfactory, and searching for an effective treatment method for patients suffering from AD is a major challenge. In the present study, we confirmed the hypothesis that repeated EA stimulation improved cognitive functioning and reduced the accumulation of Aβ_1-42_ deposits in the brain. Moreover, the beneficial effects accompanied with the up-regulation of BDNF expression and promotion of neurogenesis in the APP/PS1 double Tg mice, suggest that EA may be a promising treatment for AD.

In the last two decades, several kinds of genetically modified mice have been generated as potential models for studying neurodegenerative processes [16], such as PDAPP mice reported by Masliah et al. [17] and Tg2575 mice reported by Shi et al. [18]. In the current study, we chose the model of APP/PS1 double Tg mice, which were generated by knocking identified familial AD and/or PS1 into the mice genome; this model has been widely adopted by other scientists. The APP/PS1 double Tg mouse was used as the AD model for its aggressive, early-onset brain amyloidosis, as well as the concurrent atrophy and substantial cell loss. The clinical relevance of this model is supported by disturbances of neuronal structure in the form of dystrophic neurites surrounding plaques, decreased fiber density, and synaptic dysfunction that imitates most aspects of AD brain pathology [19,20]. Thus, the APP/PS1 double Tg mouse model is the closest representative of AD pathology. In this study, the effectiveness of the Tg mouse model to mimic AD was evaluated through neurological behavior and pathology assessment. Cognitive impairment presented in 7-month-old double Tg mice, and the Aβ deposits in the hippocampus and cortex were detected in 10-month-old double Tg mice. These findings indicate that the pathological features of APP/PS1 double Tg mice that mimic AD remained stable.

Classic and well-known symptoms of AD include problems with spatial learning and presence of a memory deficit. Previous studies have shown that EA stimulation can protect against neuronal damage, and effectively prevent the impairment of learning and memory caused by cerebral ischemia injury or high-sustained positive acceleration (+Gz) exposures [9,21]. Our study demonstrated that EA stimulation significantly restored spatial learning and memory function of AD mice. This suggests that that EA stimulation may be effective in potentially ameliorating cognitive impairment caused by AD.

The identification of reliable biomarkers has been hindered by the fact that the diagnosis of AD in clinical practice depends largely on a patient’s symptoms. However, increasingly accurate pathological diagnostic methods have become a reality because of the identification of biomarkers such as APP, Aβ, tau and p-tau, isoprostanes, and inflammatory makers. Among these, Aβ deposits are the most typical pathological sign and a defining factor for cognitive impairment in AD brains. In the brain, two different kinds of Aβ exist (Aβ1-40 and Aβ1-42). Aβ1-42, the metabolite of the APP and PS1 gene mutation, is a major component of senile plaques [22-24]. Additionally, the accumulation of Aβ1-42 in the brain is more cytotoxic than Aβ1-40, in the context of AD. Aβ is not only a biomarker for diagnosing AD; it is also an important target for AD therapy. Application of an antagonist of Aβ has been shown to improve memory impairment in APP Tg mice [25]. Thus, researchers are focusing on finding effective methods to lower Aβ deposits, especially those of Aβ1-42, to reverse the pathological features of AD. In our study, we demonstrated that EA treatment was a feasible and effective way for lowering Aβ1-42 deposits in APP/PS1 mice. However, the mechanism for EA to reduce Aβ deposit is still unclear and needing further exploration.

The subventricular zone of the lateral ventricle and the subgranular layer of the dentate gyrus in the hippocampus are two parts associated with adult neurogenesis. Dramatic decline in neurogenesis has been proven to contribute to the impairments of learning and memory in AD models [26,27]. Some key proteins involved in AD pathogenesis, including apolipoprotein E4 (APOE4), APP and PS1, have been proved to regulate neurogenesis [28-30]. Regenerative medicine, which is different from slowing down or stopping the progression of AD, may offer a new therapeutic strategy for patients. The newborn neurons may enhance neuronal plasticity and integrate into existing neural circuits physically and functionally [4]. Our results revealed that with BrdU staining, repeated EA treatment could enhance neurogenesis in APP/PS1 Tg mice. The decrease in Aβ1-42 that we mentioned above may be an important factor for nerve regeneration.

The mammalian neurotrophin family, including nerve growth factor, BDNF and neurotrophin-3, activates different cell signaling pathways via tyrosine receptor kinase. BDNF localizes in the hippocampus, hypothalamus, cortex, septum and the adrenergic brain stem nuclei of the brain and participates in neuron development, differentiation and plasticity maintenance throughout life [31]. BDNF plays an important role in memory formation and storage by regulating synaptic plasticity. Even if there have been conflicting results regarding the expression of BDNF in AD patients [32], most of the results indicate that BDNF is severely decreased in the hippocampus and some cortical areas [7,33]. Meanwhile, BDNF could reduce cellular damage caused by Aβ1-42 [34]. Our results showed that EA treatment significantly increased the expression of BDNF in both the hippocampus and cortex; this suggests that the increase in BDNF may be involved in the therapeutic effect of EA for AD. From our current study, the expression of Aβ and BDNF changing were also observed in wild-type mice in. We assumed that the effect of EA on Aβ and BDNF could be related to a common signaling pathway but not a specific pathway in AD. Moreover, the control group was age-matched with the APP mice. Our result might suggest that electroacupuncture could induce the protective effect for the central nervous system of aging and Alzheimer’s disease.

In the present study, we observed that EA stimulation significantly improved the neurological behavior performance of AD mice, and reduced the deposition of Aβ in the hippocampus and cortex. At the same time, a noticeable increase in neurogenesis and BDNF expression in the hippocampus and cortex was also detected. Thus, our preliminary presumption was that EA stimulation improved neurobehavioral performance through promoting neurogenesis and BDNF expression in the brain. However, the link between neurogenesis and the pathology of AD is still not fully understood. It is proven that Aβ deposits impair the transduction path activated by BDNF such as Ras/ERK, PI3K/Akt and PKA/CREB [35,36]. Moreover, BDNF is required for long-term survival of newborn neurons [37]. Presumably, EA may affect BDNF and its downstream pathway to induce neurogenesis, and improve neurobehavior in an AD model. The precise mechanism of EA treatment for AD still needs further elucidate.

In addition, Baihui (GV20) acupoint belongs to the Du series. According to traditional Chinese medical theory, acupoint of the Du series is the first choice in treating brain disease. Our previous experimental studies [9-11,38] have proved that EA at the Baihui acupoint protects brain from ischemic injury. Thus, we chose the Baihui acupoint in the present study. Moreover, our previous study [14] also investigated the specificity of Baihui acupoint in a rat cerebral ischemic model. Although in the current paper we aimed to focus proving that EA owned the possibility for improving cognitive dysfunction, the specificity for EA treatment in AD model still need further exploration. This is a limitation of our study, a sham-acupuncture considered as a control is necessary for future study.

## Conclusions

In summary, the present study demonstrated that EA stimulation in Baihui (GV 20) acupoint ameliorated learning and memory deficits and reduced Aβ42 deposit in APP/PS1 mice. We propose that the underlying mechanism of EA may be related to the promotion of neurogenesis and the up-regulation of BDNF expression in the hippocampus and cortex. These findings provide strong evidence for EA as a novel strategy for AD treatment.

## Abbreviations

EA: Electroacupuncture; APP/PS1: Amyloid precursor protein/presenilin 1; AD: Alzheimer’s disease; Aβ: Amyloid β-peptide; BDNF: Brain-derived neurotrophic factor; BrdU: Bromodeoxyuridine; Tg: Transgenic; ELISA: Enzyme-linked immunosorbent assay; MWM: Morris water maze.

## Competing interests

The authors have no competing interests to declare.

## Authors’ contributions

XL acquired the data, analyzed and interpreted the data and drafted the manuscript. FG drafted the manuscript. QZ carried out the Morris water maze test. TH participated in the immunofluorescence assays. LL participated in drafting the manuscript. HW carried out the western blot test. QW handled funding and supervision, analyzed and interpreted the data, performed statistical analysis and drafted the manuscript. LX conceived and designed the research, handled funding and supervision and made critical revision of the manuscript for important intellectual content. All authors read and approved the final manuscript.

## Pre-publication history

The pre-publication history for this paper can be accessed here:

http://www.biomedcentral.com/1472-6882/14/37/prepub
